# Intelligent Responsive Nanostructures Toward Food Preservation: Synergizing Antibacterial Activity and Environmental Regulation

**DOI:** 10.3390/foods15162837

**Published:** 2026-08-14

**Authors:** Jie Yu, Hao Li, Yucheng Zou, Liuyang Qiu, Xinxin Wu, Xinai Zhang

**Affiliations:** School of Food Science and Engineering, Jiangsu University, Zhenjiang 212013, China

**Keywords:** intelligent responsive nanostructures, active food preservation, antibacterial mechanisms, microenvironmental regulation, smart packaging

## Abstract

Global food spoilage precipitates monumental economic deficits and persistent public health risks, exposing the acute vulnerabilities of conventional passive preservation modalities reliant on energy-intensive refrigeration and synthetic additives. Intelligent responsive nanostructures (IRNs) offer a transformative paradigm for active food preservation. This review elucidates how IRNs transcend conventional approaches through dual modulation of targeted antibacterial activity and microenvironmental dynamics. Freshness visualization technologies based on colorimetric and fluorescent responses to spoilage biomarkers are delineated. Subsequently, the hierarchical antibacterial mechanisms of IRNs are elaborated, encompassing physical membrane disruption, controlled ion release, reactive oxygen species (ROS) generation, and genetic interference. Crucially, we emphasize the synergistic strategies where lethal mechanisms are coupled with environmental regulation. The construction of active prevention systems is presented, alongside critical biosafety challenges toward sustainable nanoplatforms that autonomously maintain food quality from farm to fork.

## 1. Introduction

According to the Food and Agriculture Organization (FAO) of the United Nations, approximately 1.3 billion tonnes of food are wasted annually [1,2,3]. Among the various contributors to food deterioration [4,5,6], microbial contamination stands as the most pervasive and destructive factor [7,8,9]. Pathogenic bacteria such as *Salmonella* [10,11], *Escherichia coli* [12,13], and *Listeria monocytogenes* [14] not only accelerate spoilage but also pose severe threats to public health. As food supply chains become increasingly globalized, the interplay of microbial contamination with chemical residues and physical damage has driven a 5% annual rise in foodborne disease incidence. The alarming statistics underscore the critical importance of antibacterial intervention in food preservation [15,16,17,18]. Therefore, effective antimicrobial patterns are essential not merely for extending shelf life but for preventing the proliferation of harmful microorganisms [19,20,21,22].

The traditional “post-event prevention and control” model, heavily reliant on terminal sampling and laboratory testing [23,24,25], is inadequate for addressing the real-time risks inherent in food supply chains. This reactive approach depends predominantly on passive antibacterial strategies [26,27,28,29], such as refrigeration and chemical preservatives [30,31]. Specifically, refrigeration merely slows microbial metabolism rather than eliminating pathogens [32,33], in which even minor temperature fluctuations can rapidly reactivate bacterial growth; likewise, chemical preservatives face growing consumer resistance due to toxicity concerns, and are often ineffective against biofilm-forming or multidrug-resistant strains [34,35]. Moreover, the conventional methods lack the ability to sense or respond to changing contamination levels [36]; they apply a fixed intervention regardless of actual microbial load. Consequently, microbial proliferation triggered by subtle temperature deviations frequently goes undetected until products arrive at terminal markets.

The emergence of intelligent responsive nanostructures (IRNs) marks a transformative shift from “passive testing” to “active prevention and control” in food preservation [37,38,39]. The advanced nanostructures possess the unique capacity to dynamically sense environmental changes and execute targeted responses in real time [40]. Through the responsiveness to external stimuli, IRNs can autonomously sense early signs of food deterioration. Upon capture, they activate built-in antibacterial behavior, thereby suppressing pathogen proliferation at its onset [41,42]. The dual capability enables the establishment of a self-regulating quality assurance that spans the entire food lifecycle, from harvest and processing to storage, transport, and retail display.

To eliminate any ambiguity regarding our core subject, IRNs are defined in this review as advanced [43], multi-functional nano-architectures that integrate localized physicochemical sensing, autonomous decision-making (via stimulus-triggered mechanisms), and active intervention (hierarchical antibacterial action and microenvironmental regulation) within a unified platform. To precisely contextualize IRNs, they must be clearly distinguished from several related categories: First, unlike conventional nanomaterials that operate via static physical or chemical properties without environmental feedback, IRNs possess dynamic responsiveness. Second, compared to biomedical stimuli-responsive nanomaterials that target human pathological triggers, IRNs are specifically engineered for food spoilage kinetics, reacting directly to foodborne biomarkers (e.g., TVB-N [44], biogenic amines [45,46], and H_2_S [47,48]). Third, while smart packaging systems are primarily designed for external perception and visual/digital communication [49], IRNs provide the active nanoscale execution units capable of direct remediation. Fourth, unlike active packaging which typically relies on continuous or un-triggered release/scavenging, IRNs achieve a “sense-and-act” mechanism, releasing active agents or regulating microenvironments strictly on-demand when spoilage cues reach critical thresholds. External digital frameworks (e.g., IoT and blockchain) serve as complementary macroscopic data infrastructures that digitize signal outputs generated by these physical IRN units.

**Scheme 1:**
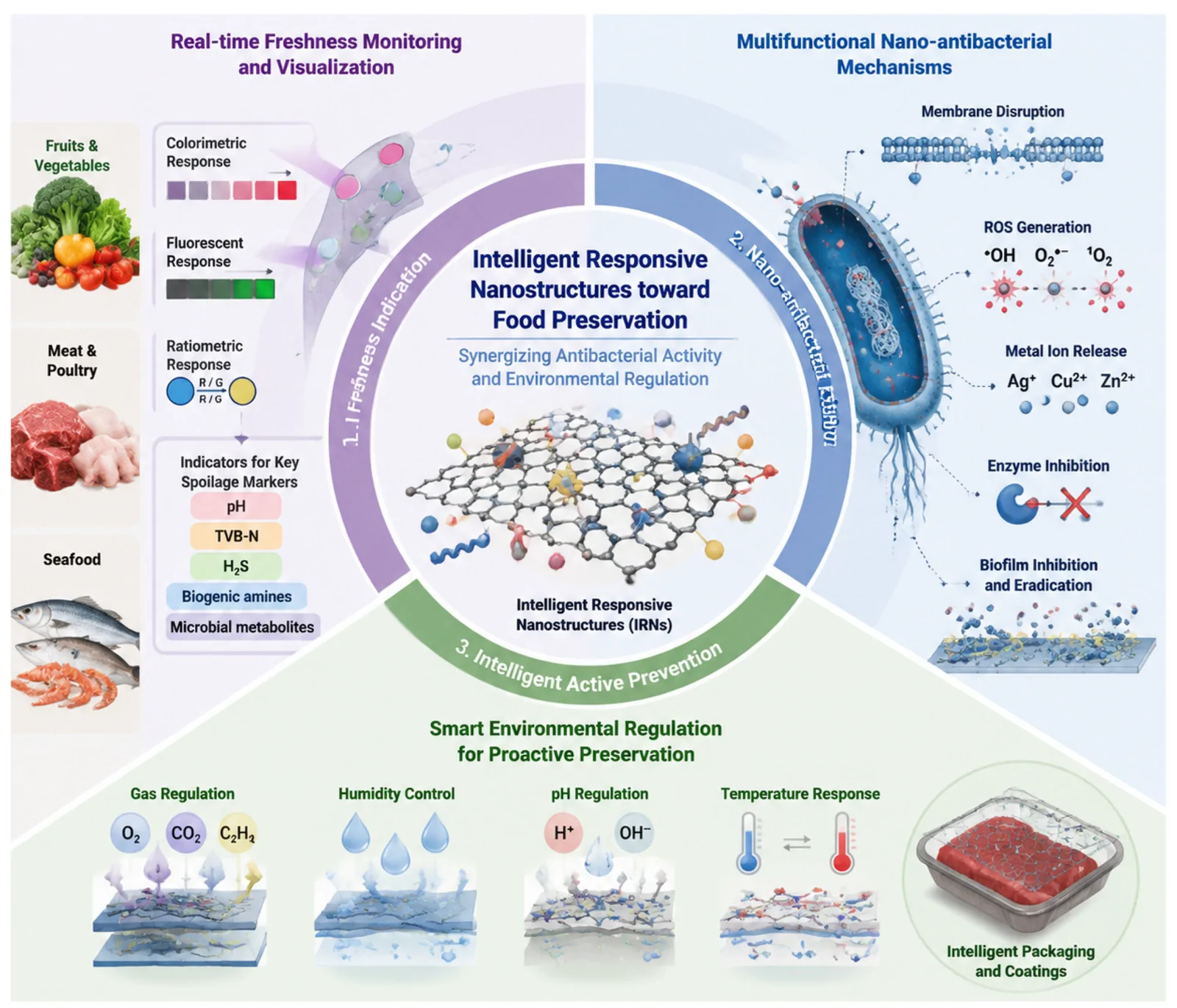
Schematic diagram of intelligent responsive nanostructures toward active food preservation.

This review provides a comprehensive overview of the recent advances in IRNs for active food preservation. We begin by introducing IRN-based freshness indication that enable visual awareness of spoilage biomarkers via colorimetric or fluorescent responses (Scheme 1). The hierarchical antibacterial mechanisms of IRNs are then elaborated, followed by a discussion of how the self-adjusting preservation microenvironments can be created. Furthermore, the construction of active prevention systems integrating IRNs into packaging, cold chain logistics, and traceability platforms is examined. Finally, biosafety considerations, multifunctional precision design, and the prospects of AI-IoT-enabled sustainable nanoplatforms are discussed, offering insight into the transition from passive to autonomous food quality assurance.

**Scheme 2:**
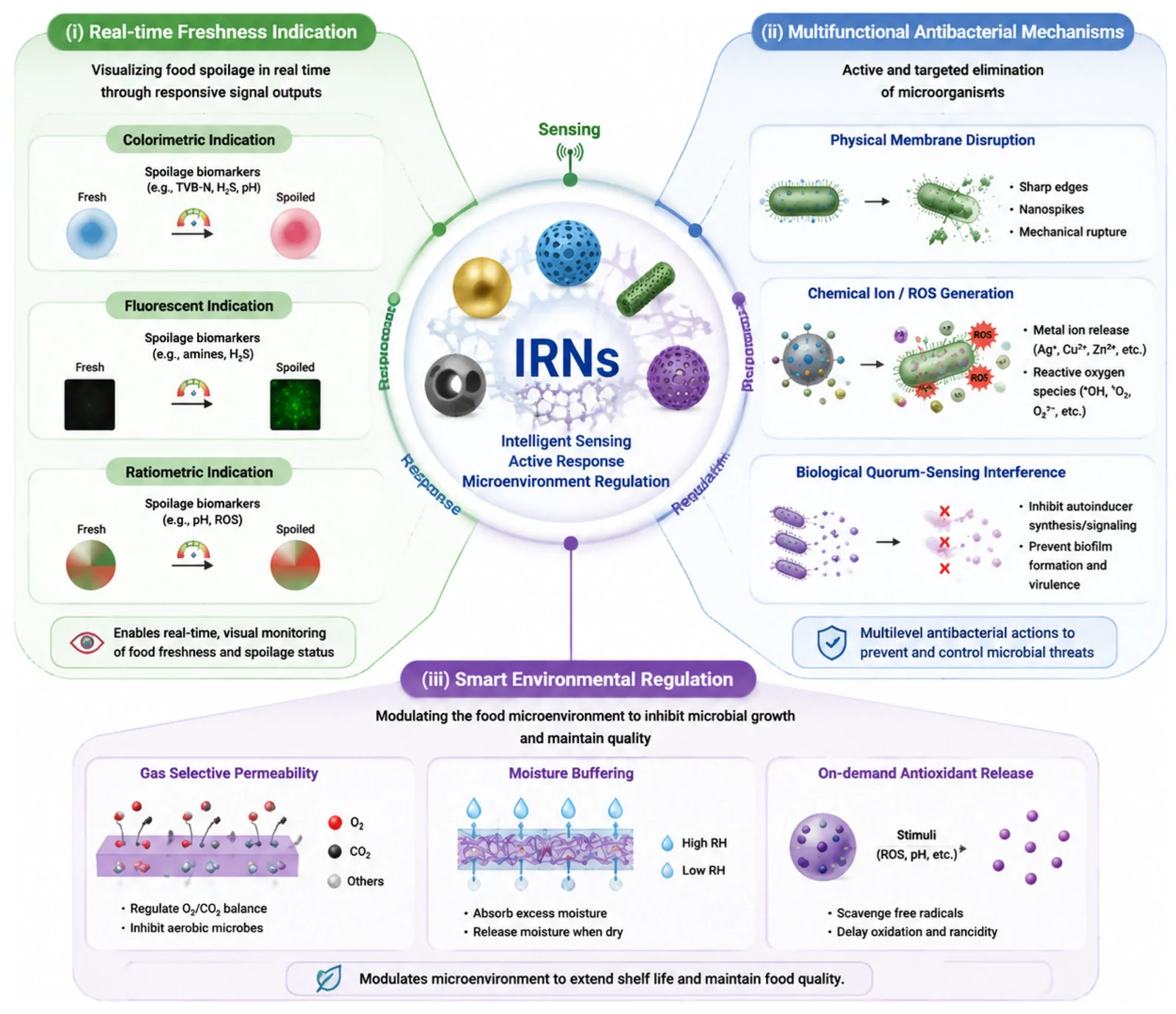
Schematic illustration of the classification of IRNs based on freshness indication, hierarchical antibacterial mechanisms, and smart environmental regulation.

To provide a clear structural framework for this review, Scheme 2 illustrates the comprehensive classification of IRNs based on their functional modalities. Specifically, IRN-based systems are systematically categorized into three main branches: (i) Real-time Freshness Indication (encompassing colorimetric, fluorescent, and ratiometric signal outputs responding to spoilage biomarkers); (ii) Multifunctional Antibacterial Mechanisms (spanning physical membrane disruption, chemical ion/ROS generation, and biological quorum-sensing interference); (iii) Smart Environmental Regulation (including gas selective permeability, moisture buffering, and on-demand antioxidant release). This multi-dimensional classification highlights how IRNs synergistically bridge sensing and active preservation.

Despite the growing body of literature on intelligent food packaging and nanomaterials [50,51], a critical knowledge gap persists in the current field. Most existing reviews tend to treat freshness indication, antimicrobial action, or environmental control as isolated, open-loop phenomena, focusing either narrowly on specific carrier materials or on macroscopic supply chain tracking without addressing the underlying nanoscale mechanisms. Furthermore, the systematic integration of a “sense-response-execution” framework, where structural transformations driven by spoilage biomarkers simultaneously trigger precise antibacterial defense and microenvironmental optimization, has not been thoroughly summarized. To bridge this gap, the present review provides a holistic multi-dimensional taxonomy of IRNs, bridging microscopic hierarchical antimicrobial pathways with macroscopic AI-IoT-enabled cold chain systems. Table 1 explicitly compares recent reviews with the distinctive contributions of this work.

## 2. Nanomaterial-Based Freshness Indication

**Figure 1:**
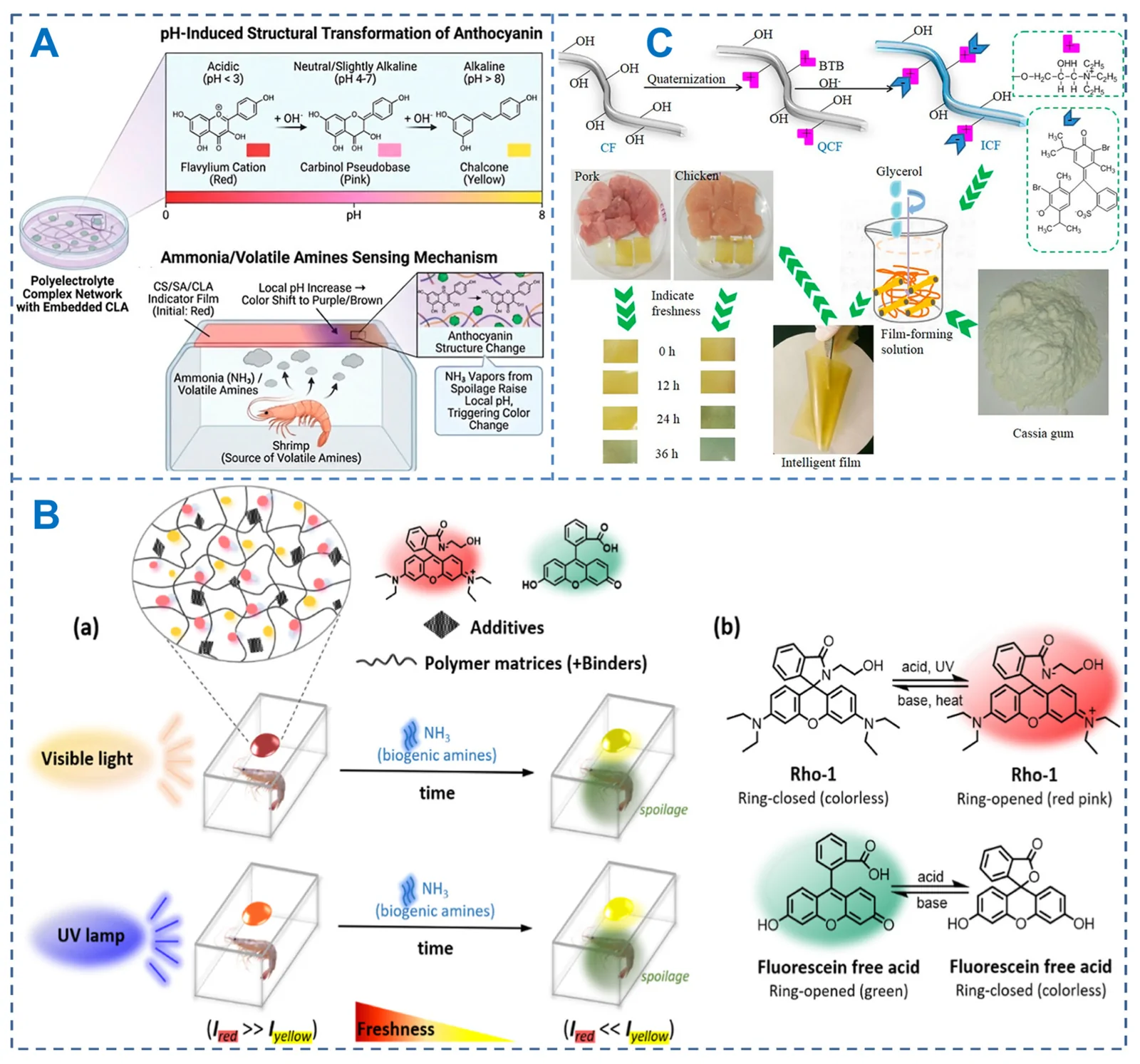
(**A**) Chitosan/sodium alginate composite intelligent film incorporating *Campanumoea lancifolia* anthocyanins for monitoring shrimp freshness. Reprinted with permission from Ref. [61]. Copyright 2026 American Chemical Society. (**B**) Dual-color ratiometric fluorescent sensors for highly sensitive detection of food spoilage. Reprinted with permission from Ref. [62]. Copyright 2023 American Chemical Society. (**C**) Intelligent film based on cassia gum containing bromothymol blue-anchored cellulose fibers for real-time detection of meat freshness. Reprinted with permission from Ref. [63]. Copyright 2019 American Chemical Society.

Nanomaterial-based freshness indication leverages the response of IRNs to stimuli generated during food spoilage [55,56,57]. The sensing patterns integrate nanomaterials with indicator molecules to produce visual signal outputs, including colorimetric transitions and fluorescence variations [58,59,60]. By embedding the indicators directly into packaging materials, conventional passive packages are transformed into intelligent sensing platforms capable of communicating real-time quality information (Figure 1), thereby enabling proactive intervention before spoilage becomes visually apparent.

### 2.1. Response Patterns for Freshness Indication

Color change remains the most intuitive response in nanomaterial-based freshness indication [64,65,66,67,68,69]. The sensing principle relies on the optical response of IRNs to microbial stimuli such as TVB-N [70,71,72], H_2_S [47], and pH variations [73,74,75,76]. For example, intelligent packaging films incorporating natural anthocyanins exhibit pronounced color transitions across the visible spectrum in response to pH changes [77,78]: they appear red under acidic conditions (pH 1~4), transition to a colorless methanol pseudobase at pH 4~6, form pink-to-purple quinonoid bases at pH 6~8, and generate yellow chalcones at pH above 8. Similarly, curcumin-based indicators shift from yellow to reddish-brown as ammonia accumulates [79,80], providing a clear visual cue for protein-rich food spoilage (Figure 1A). The colorimetric responses enable instantaneous freshness assessment without specialized equipment [81,82,83,84], making them highly suitable for consumer-facing applications.

Fluorescence signal offers superior sensitivity compared to colorimetric methods, enabling evaluation of spoilage biomarkers at low levels [85,86,87]. The sensing mechanism is based on specific interactions between spoilage biomarkers and photophysical properties of fluorescent nanomaterials (e.g., quantum dots (QDs) [88,89], metal nanoclusters [90], aggregation-induced emission (AIE) luminogens [91], and upconversion nanoparticles [92]). The signal probes undergo specific binding or chemical reactions with VOCs [93], biogenic amines [94], or H_2_S [95] released during spoilage [48], resulting in fluorescence enhancement (turn-on), quenching (turn-off), or wavelength shift (ratiometric). Ratiometric fluorescent sensors [96,97], which measure the ratio of two emission peaks (Figure 1B), offer built-in self-calibration that minimizes interference from background signals and excitation fluctuations, making them particularly advantageous for food matrices where multiple interfering substances coexist.

A critical comparison of colorimetric and fluorescent sensing systems reveals distinct performance trade-offs that dictate their suitability for practical food monitoring (Table 2). While fluorescent sensors, typically utilizing quantum dots, carbon dots, or organic fluorophores exhibit exceptionally low detection limits and high sensitivity toward trace spoilage metabolites, they are frequently constrained by photobleaching, susceptibility to optical quenching in complex food matrices, and the requirement for UV–Vis excitation equipment, which hinders consumer-facing commercial adoption. Conversely, colorimetric systems driven by plasmonic metal nanoparticles or halochromic dyes offer intuitive, naked-eye visual readouts and rapid response times, making them exceptionally promising for low-cost, smart packaging. However, colorimetric sensors often struggle with limited long-term stability due to dye leaching or oxidation, restricted reversibility, and lower quantitative accuracy. Balancing these parameters is crucial for future commercial deployment, where cost-effectiveness and user-friendly visual interpretation must be weighed against analytical precision.

### 2.2. Intelligent Indicator Labels

The intelligent indicator labels center on key biomarkers associated with food spoilage [104], exploiting the tunable properties of IRNs to construct a functional system integrating signal perception, transduction, and visual output (Figure 1C). First, the sensing unit requires careful selection of specific responsive materials based on the spoilage characteristics of different food categories. For ethylene (C_2_H_4_) gas released during the respiration of fruits [105,106,107], porous MOFs serve as adsorption carriers and signal transducers [52,108,109], enabling early-stage ripeness monitoring before overt spoilage occurs. For biogenic amines produced during the decomposition of meat products [110,111], pH-responsive nanomaterials (e.g., anthocyanin-loaded gelatin [112], polyaniline nanocomposites [113]) can provide rapid and reversible colorimetric responses. Additionally, for CO_2_ accumulation in modified atmosphere packaging [114], amine-functionalized nanomaterials can trigger distinct color changes proportional to CO_2_ concentration.

Moreover, intelligent indicator labels should also prioritize the integration of multiple response mechanisms and expansion of intelligent functionalities [115,116,117]. Composite films incorporating both colorimetric and fluorometric elements can simultaneously detect multiple spoilage markers, enhancing accuracy and reducing false positives. Furthermore, incorporation of smartphone-readable Quick Response (QR) codes or Near Field Communication (NFC) tags enables digital recording and sharing of freshness data [118,119,120]. The substrate material (e.g., cellulose paper [121,122], biodegradable polymer film [123], or edible coating [124]) are selected to ensure compatibility with food contact, mechanical robustness during handling, and stable performance under varying temperature and humidity. Advanced designs may also incorporate time–temperature integrators (TTIs) to monitor cumulative thermal abuse throughout distribution [125], providing a comprehensive picture of product history beyond instantaneous freshness assessment.

## 3. Antibacterial Mechanisms of Irns

**Figure 2:**
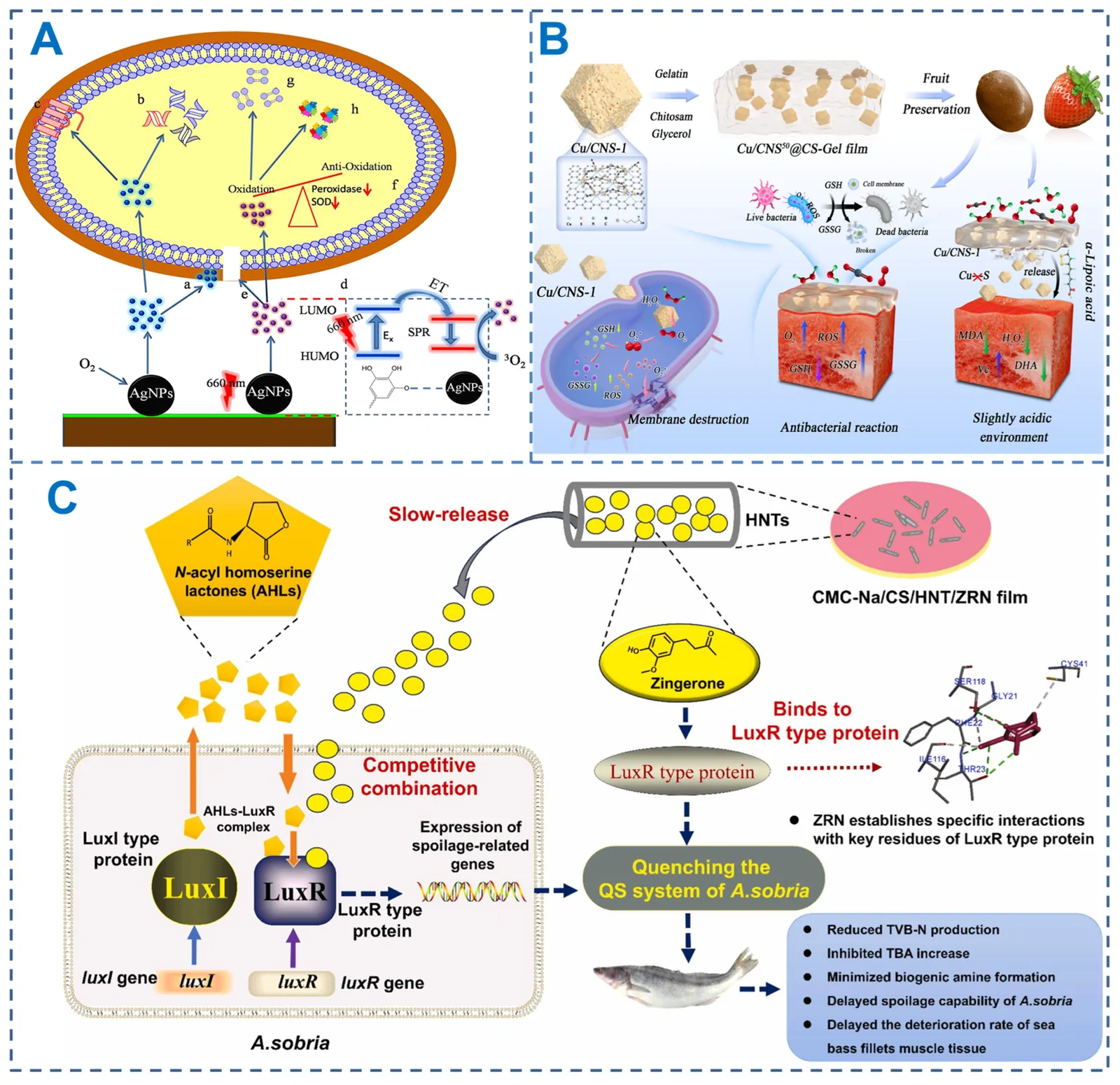
(**A**) Schematic illustration of the mechanism of physical antibacterial activity of TA/Fe^3+^/AgNPs. Reprinted with permission from Ref. [126]. Copyright 2017 American Chemical Society. (**B**) Silica-coated upconversion nanoplatform for Ag-based chemo-/photodynamic therapy against drug-resistant bacteria. Reprinted with permission from Ref. [127]. (**C**) Inhibition mechanism of slow-release zingerone in CMC-Na/CS/HNT/ZRN film on *A. sobria* spoilage in sea bass fillets. Reprinted with permission from Ref. [128]. Copyright 2025 Elsevier.

The antibacterial mechanisms of IRNs operate through a hierarchical framework that distinguishes them from conventional single-mode antimicrobial agents. IRNs integrate physical, chemical, and biological antibacterial pathways that can be activated in response to specific spoilage triggers (Figure 2).

### 3.1. Physical Antibacterial Mechanisms

Physical antibacterial mechanisms exploit the unique size effects, morphological features (Figure 2A), and surface properties of IRNs to directly compromise microbial structural integrity [129,130]. Physical mechanisms act immediately upon contact [131,132,133,134], providing rapid initial suppression of microbial proliferation. Among these mechanisms [135], membrane disruption represents the most prevalent and fundamental pathway. When nanomaterials contact microbial cell membranes [136,137], their nanoscale dimensions and high specific surface area facilitate strong adsorption onto membrane surfaces via electrostatic or hydrophobic interactions. This adsorption induces localized membrane thinning, pore formation, and eventual loss of barrier function, leading to cytoplasmic leakage and cell death.

Mechanical damage constitutes another critical physical mechanism [27,138], wherein nanomaterials with sharp or anisotropic morphologies (such as nanofillers [89,139], nanofibers [140,141], nanowires [142], and nanoparticles [143]) physically puncture or lacerate microbial cell walls and membranes upon contact. The bactericidal efficiency of this mechanism is highly dependent on the orientation, density, and rigidity of the nanostructures relative to the target cell surface. Notably, the physical damage inflicted by IRNs creates localized disruptions that facilitate subsequent penetration of released metal ions or reactive species [144], establishing a synergistic cascade between physical and chemical antibacterial pathways. This physical–chemical synergy is particularly valuable for combating biofilm-embedded pathogens [145], where conventional antibiotics often fail due to limited penetration.

### 3.2. Chemical Antibacterial Mechanisms

Chemical antibacterial mechanisms involve the release of bioactive ions or generation of reactive species that interfere with microbial biochemical processes [146,147] (Figure 2B). Ion release is a core pathway, primarily involving metal-based IRNs (e.g., ZnO [148,149], and TiO_2_ [150]) that gradually release metal ions into the surrounding microenvironment in response to pH changes, temperature elevation, or enzymatic activity characteristic of spoilage progression. Silver ions (Ag^+^) bind strongly to thiol groups in microbial enzymes and structural proteins [151], disrupting respiratory chains and ATP synthesis. They also interact with DNA, preventing replication and transcription, thereby arresting cell division. Zinc ions (Zn^2+^) inhibit protease activity and interfere with protein synthesis [152,153], while copper ions (Cu^2+^) promote Fenton-like reactions that generate additional oxidative stress [154]. The controlled, stimulus-triggered release profile of IRNs ensures sustained antibacterial activity over extended storage periods while avoiding the burst-release toxicity associated with free metal salts.

Reactive oxygen species (ROS) generation represents another potent chemical mechanism [155,156], wherein IRNs catalyze the production of hydroxyl radicals (•OH), superoxide anion radicals (O_2_^−^), and hydrogen peroxide (H_2_O_2_) through photocatalytic, sonodynamic, or chemodynamic processes. The ROS are powerful oxidizing agents that non-selectively attack lipids, proteins, and nucleic acids [157,158], causing irreversible damage to microbial cell membranes and intracellular components. Importantly, the ROS-generating activity of many IRNs can be modulated by external stimuli—for instance, UV or visible light activation of photocatalytic nanomaterials [159,160]—or acidic pH triggering of peroxidase-mimicking nanozymes [161], allowing spatial and temporal control over antibacterial action. The stimulus-responsive ROS generation aligns perfectly with the “active prevention” paradigm, where antibacterial activity intensifies precisely when spoilage conditions develop.

### 3.3. Biological Antibacterial Mechanisms

Biological antibacterial mechanisms target microbial regulatory networks and metabolic pathways at the molecular level [162,163,164,165], offering highly specific interference with minimal impact on food matrix components (Figure 2C). A prominent strategy involves mimicking key enzyme inhibitors in microbial metabolic pathways. Through competitive binding or irreversible inhibition, certain IRNs can block core metabolic routes such as the tricarboxylic acid cycle [166], fatty acid biosynthesis [167], or cell wall peptidoglycan synthesis [168]. For example, metal oxide nanoparticles can coordinate with essential cofactors in metalloenzymes, displacing native metal ions and rendering the enzymes catalytically inactive.

Interference with microbial quorum sensing (QS) systems represents another sophisticated biological mechanism [169,170,171]. QS is a cell density-dependent communication process through which bacteria coordinate virulence factor expression, biofilm formation [172,173,174], and antibiotic resistance development. IRNs can disrupt QS in multiple ways: AgNPs bind to and degrade autoinducer signaling molecules (e.g., acyl-homoserine lactones in Gram-negative bacteria) [175]; ZnO nanoparticles downregulate QS-related gene expression [176]; and polymeric nanoparticles encapsulating QS inhibitors [172] provide sustained release for prolonged anti-virulence effects. By disabling bacterial communication without directly killing cells [177], this mechanism reduces selective pressure for resistance while attenuating pathogenicity. When combined with physical and chemical mechanisms, QS interference creates a multilayered defense that prevents biofilm maturation and maintains bacterial populations below spoilage thresholds throughout the food supply chain.

Table 3 compares the physical, chemical, and biological antibacterial mechanisms of IRNs across key parameters. The comparison highlights how each mechanism possesses distinct advantages and limitations, while their synergistic interactions collectively enable a multi-hit strategy that overcomes the shortcomings of any single approach.

While classifying the antibacterial action of IRNs into physical, chemical, and biological categories provides a useful pedagogical framework, it inherently oversimplifies the complex, multi-hit behavior of advanced nanomaterials. In practical food preservation scenarios, most metal-based or multifunctional IRNs do not act through isolated pathways; rather, these mechanisms operate in a tightly coupled, cascading network. For instance, the initial physical adsorption and localized membrane disruption caused by high specific surface areas and sharp morphologies create nanoscale pores that drastically lower the diffusion barrier for metal ions (Ag^+^, Zn^2+^, etc.). Once internalized, these ions disrupt respiratory enzymes and synergistically trigger Fenton-like or photocatalytic generation of ROS [180]. This overwhelming oxidative stress subsequently inflicts irreversible damage on intracellular nucleic acids and proteins, which concurrently interferes with metabolic pathways and quorum-sensing communication. Recognizing these dynamic overlaps is essential, as this multi-target synergy minimizes the risk of bacterial resistance and drives the high-efficiency sterilization observed in advanced IRN systems.

### 3.4. Classification of Irn Response Mechanisms

To fully capture the versatility of IRNs, their operation can be systematically classified according to the specific environmental triggers driving their “sense-and-act” behavior, encompassing: (1) pH-responsive systems activated by microbial acid or volatile amine accumulation [181,182,183]; (2) temperature-responsive systems utilizing thermo-responsive polymers or phase-change materials to buffer cold-chain excursions [184,185]; (3) humidity-responsive systems driven by relative humidity gradients to prevent condensation and dehydration [186]; (4) enzyme-responsive system employing cleavable linkers activated by spoilage-related enzymes [187]; (5) gas-responsive systems designed to capture or react with key gases via porous nanostructures [188]; (6) light-responsive systems utilizing photocatalytic platforms to generate reactive oxygen species upon illumination [189,190]; (7) mechanically responsive systems exploiting physical stress- or pressure-induced deformations during handling to trigger smart release or signals [191].

## 4. Intelligent Preservation

**Figure 3:**
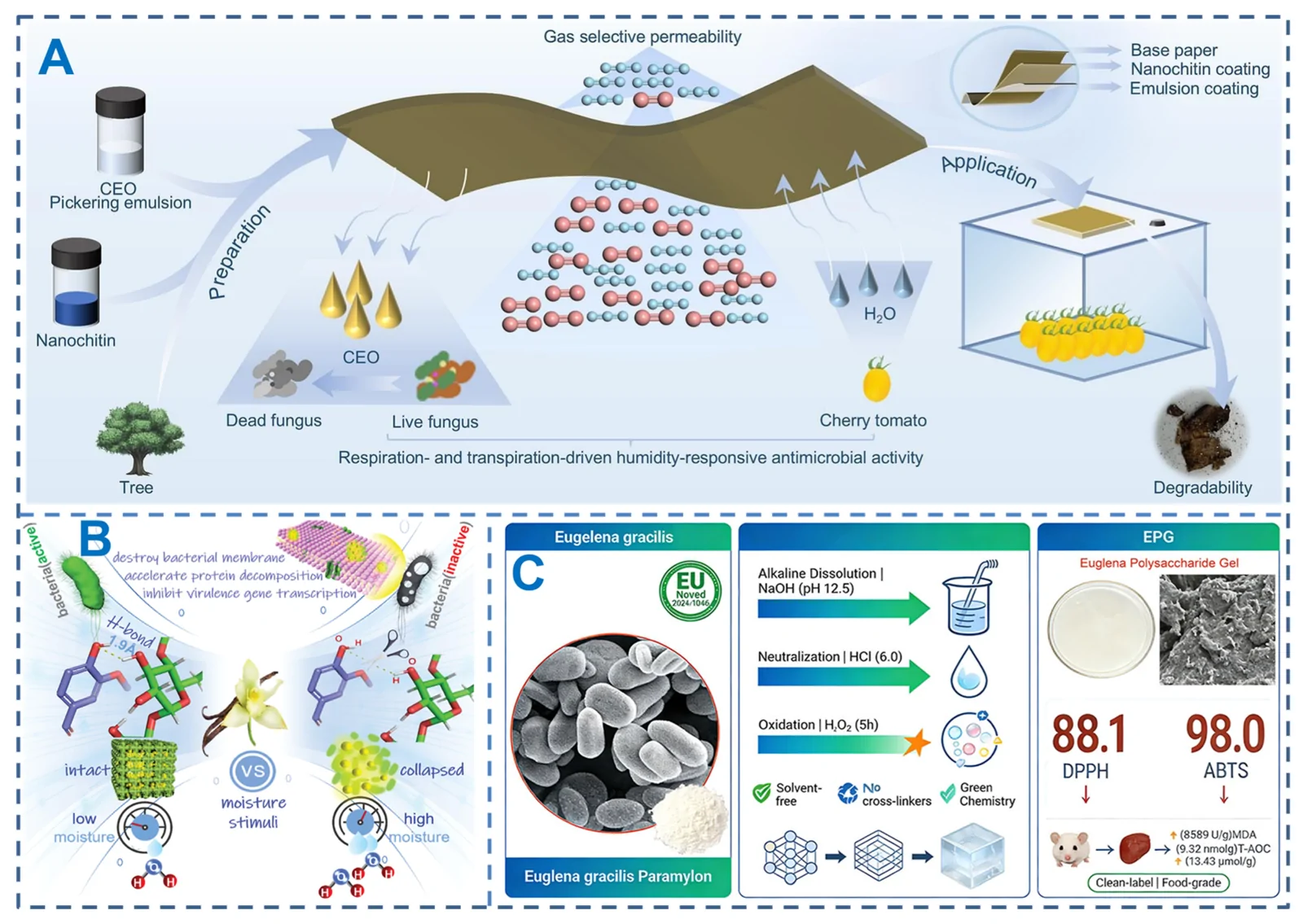
(**A**) Multilayer packaging with CO_2_/O_2_-selective gas regulation for cherry tomato preservation. Reprinted with permission from Ref. [192]. Copyright 2026 Elsevier. (**B**) Moisture-driven bacteriostat evolved from phytochemical formulation. Reprinted with permission from Ref. [186]. Copyright 2024 John Wiley and Sons. (**C**) Oxidative-gelation engineering of *Euglena gracilis* β-1,3-Glucan. Reprinted with permission from Ref. [193]. Copyright 2026 American Chemical Society.

Beyond direct antibacterial action, IRNs achieve holistic food preservation through intelligent regulation of storage microenvironment. The following subsections detail three primary regulatory modalities (Figure 3): gas regulation, moisture regulation, and antioxidant preservation.

### 4.1. Gas Regulation Preservation

Gas regulation preservation exploits the unique pore structures, surface chemistry, and stimulus-responsive properties of IRNs to dynamically control the gaseous composition within food packaging environments [194,195,196]. By modulating O_2_ [197], CO_2_ [195], and C_2_H_4_ concentrations [198], the nanomaterials delay oxidative spoilage, suppress respiration rates, and extend shelf life [199]. The regulatory mechanisms encompass adsorption, catalytic conversion, and controlled release (Figure 3A).

Specifically, reducing O_2_ concentration inhibits aerobic respiration and enzymatic browning in fresh produce [200], while elevating CO_2_ levels suppresses microbial growth and further decelerates metabolic activity. IRNs such as CeO_2_ [201] and SiO_2_ [202,203] can selectively adsorb excess CO_2_ or C_2_H_4_, releasing them only when internal concentrations drop below optimal thresholds. Alternatively, oxygen-scavenging nanoparticles regulate ROS metabolism [204] in response to humidity or temperature triggers. This intelligent, on-demand gas regulation avoids the drawbacks of passive sachets (e.g., premature exhaustion or insufficient capacity) and ensures a consistently favorable atmosphere throughout storage and distribution.

### 4.2. Moisture Regulation Preservation

Moisture regulation preservation centers on the precise control of water distribution [205,206,207,208], and phase state within foodstuff using the high specific surface area, porous architectures, or responsive hydrophilic/hydrophobic groups of IRNs (Figure 3B). Excessive moisture accelerates microbial proliferation and enzymatic hydrolysis [209], whereas excessive dehydration causes textural degradation and weight loss. IRNs can adsorb, retain, release, or directionally guide moisture to maintain optimal water activity (a_w_) levels.

For fresh fruits and vegetables [210,211], humidity-responsive nanomaterials can absorb excess water vapor during high-humidity periods and release it when the headspace becomes too dry, buffering against condensation and desiccation. For meat and aquatic products, moisture-regulating coatings form a semi-permeable barrier that slows surface evaporation, while preventing water migration from the interior to the surface. By maintaining stable moisture gradients, the IRNs preserve cellular turgor, texture, and appearance, while simultaneously limiting water availability required for bacterial growth.

### 4.3. Antioxidant Preservation

Antioxidant preservation exploits IRNs that either scavenge free radicals or release natural antioxidants in response to oxidative stress signals (Figure 3C). Lipid oxidation is a major cause of rancidity in fatty foods, generating off-flavors and toxic aldehydes [212]. Conventional antioxidants suffer from rapid depletion or thermal degradation [213]; IRNs overcome the limitations through controlled, stimulus-triggered delivery.

Two principal strategies are employed. First, nano-antioxidants with intrinsic radical-scavenging activity (e.g., cerium oxide nanoparticles [214,215], selenium nanoparticles [216,217], or functional silica [218,219]) catalyze the decomposition of peroxides and neutralize ROS through multiple redox cycles. Second, carrier-type IRNs encapsulate natural antioxidants (e.g., tocopherols [220,221], chlorogenic acid [222,223], polyphenols [224,225], citric acid [226,227]) within responsive matrices that release their payload upon exposure to rising oxygen levels, increased temperature, or pH shifts. For instance, zein nanoparticles loaded with curcumin release the antioxidant when lipid hydroperoxides accumulate.

To enable an objective evaluation of IRNs [228], a quantitative analysis of their performance parameters is essential (Table 4). Representative literature data demonstrate that advanced stimulus-responsive antibacterial nanoplatforms typically achieve a 3-log-to-5-log reduction against major foodborne pathogens like *E. coli* and *S. aureus* within response times ranging from 15 min to 2 h upon triggering [229,230]. Regarding release kinetics, responsive systems exhibit minimal premature leakage (< 5% cumulative release under normal baseline conditions) but undergo rapid, on-demand burst or sustained release (reaching 70~90% release within 24~48 h) upon encountering microbial metabolites or acidic triggers [231]. This precise kinetic control translates to a significant shelf-life extension of 50% to 200% across vulnerable food matrices such as fresh meats and seafoods [232,233]. Furthermore, migration assays confirm that active component levels remain well below strict regulatory thresholds (e.g., overall migration < 10 mg/dm^2^) [234,235], ensuring safety for human consumption.

## 5. Applications in Different Food Systems

**Figure 4:**
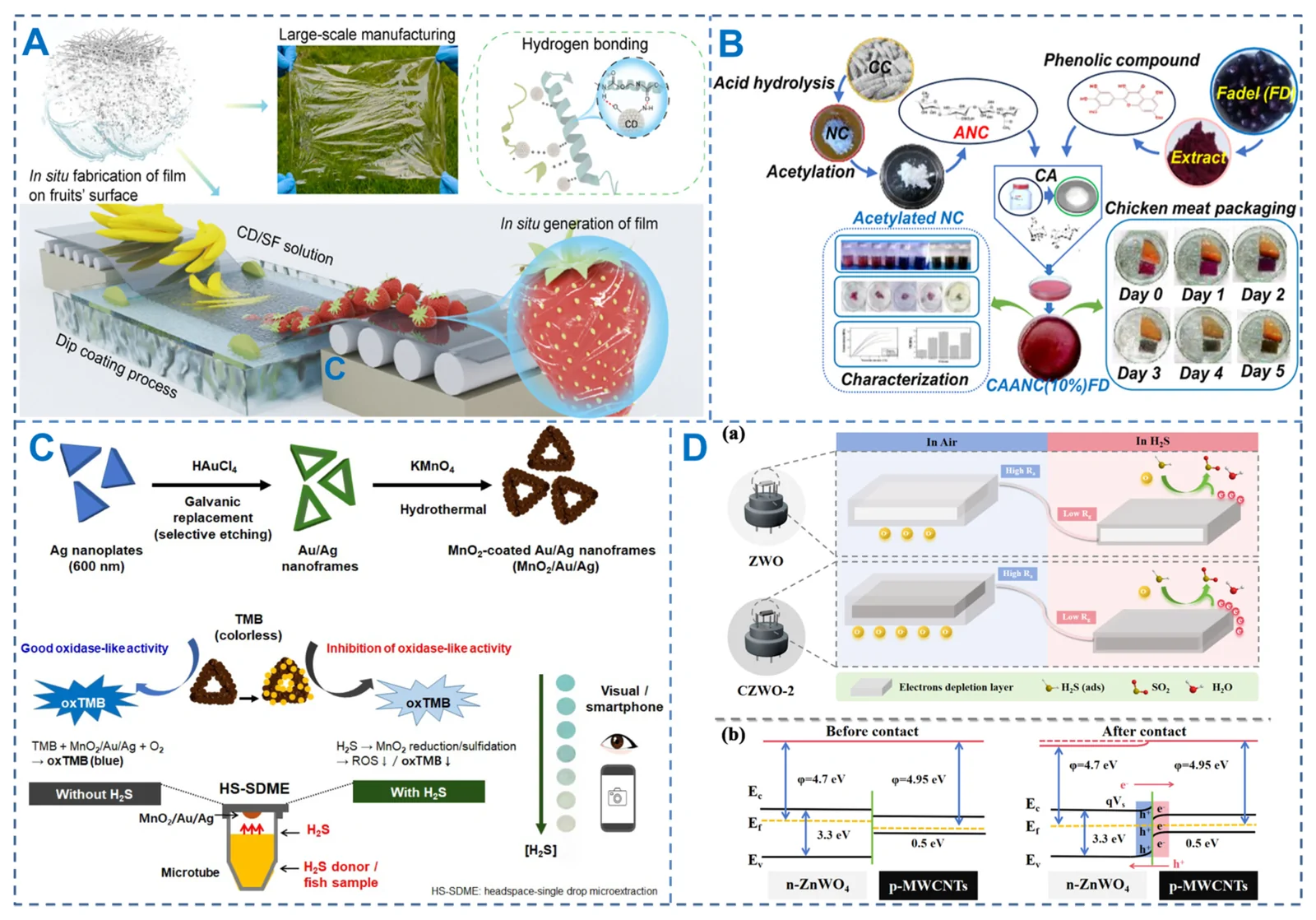
(**A**) Highly antibacterial and antioxidative carbon nanodots/silk fibroin films for fruit preservation. Reprinted with permission from Ref. [260]. Copyright 2023 American Chemical Society. (**B**) Self-synthesized acetylated nanocellulose and *Syzygium cumini* (L.) Skeels (Fadel) fruit extract-incorporated hydrophobic and intelligent film for chicken meat freshness monitoring. Reprinted with permission from Ref. [261]. Copyright 2025 American Chemical Society. (**C**) MnO_2_/Au/Ag hollow nanoframes for smartphone-assisted colorimetric detection of H_2_S during fish spoilage. Reprinted with permission from Ref. [262]. Copyright 2026 American Chemical Society. (**D**) ZnWO_4_/f-MWCNTs nanocomposite for H_2_S gas sensor for detecting fish spoilage. Reprinted with permission from Ref. [263]. Copyright 2025 American Chemical Society.

While the antibacterial mechanisms (Section 3) and environmental regulation modalities (Section 4) provide universal principles, the following subsections examine how IRNs are customized for different food categories (Figure 4).

### 5.1. Fruits and Vegetables

Fresh fruits and vegetables present unique preservation challenges due to their active post-harvest respiration [264,265,266], high susceptibility to water loss and wilting [267,268,269], and vulnerability to bacterial infections [270,271,272]. The preservation strategy centers on regulating respiration rate [273], C_2_H_4_-mediated ripening, and moisture balance through intelligent gas and humidity management (Figure 4A).

C_2_H_4_ serves as the primary ripening hormone and spoilage biomarker for climacteric fruits. IRNs incorporating porous MOFs such as ZIF-8 [274] can selectively adsorb C_2_H_4_ from the package headspace, delaying ripening and senescence. When combined with colorimetric indicators (e.g., permanganate-impregnated SiO_2_ [275]), the systems provide dual functionality: SiO_2_ removal and visual ripeness monitoring. For moisture-sensitive leafy greens and berries, humidity-responsive nanocellulose films grafted with poly(N-isopropylacrylamide) (PNIPAM) absorb excess water vapor during cold storage and release it when humidity drops, preventing condensation-induced decay and desiccation-induced shriveling. Furthermore, pH-responsive anthocyanin-loaded chitosan nanoparticles incorporated into edible coatings enable visual indication of spoilage onset through color transitions from red (fresh) to blue-green (spoiled) [276], empowering immediate freshness assessment without opening the package.

### 5.2. Meat Products

Meat products are highly susceptible to lipid oxidation [277,278,279], protein degradation [280,281], microbial contamination [282,283,284], and color deterioration [98,285]. The preservation strategy emphasizes multi-synergistic systems combining antioxidant protection [286], antibacterial action [287,288], and freshness indication [289], all triggered by spoilage-relevant stimuli such as pH elevation, oxygen ingress, and biogenic amine accumulation [290,291,292].

For lipid oxidation control [293,294,295], oxygen-responsive nano-antioxidants (e.g., α-tocopherol-loaded mesoporous SiO_2_ nanoparticles capped with enzyme-cleavable linkers) release their payload when oxygen levels rise within the package, providing on-demand protection against rancidity. Simultaneously, pH-responsive IRNs (such as curcumin-encapsulated zein nanoparticles [296]) exhibit colorimetric transitions from yellow to reddish-brown as TVB-N accumulates [297], enabling visual freshness grading (Figure 4B). For antibacterial defense, silver nanoparticle-decorated graphene oxide (AgNP-GO) composites incorporated into packaging liners release Ag^+^ in response to the acidic microenvironment created by lactic acid bacteria during spoilage, achieving targeted antimicrobial action while minimizing metal ion migration into meat tissue. The triple-function system (antioxidant release [298,299], colorimetric indication [300], and pH-triggered antibacterial activity [301,302]) exemplifies the “active prevention” paradigm, where multiple protective mechanisms are coordinated autonomously based on real-time spoilage signals.

### 5.3. Aquatic Products

Aquatic products pose the most demanding preservation challenges due to their exceptionally high moisture content, abundant free amino acids and polyunsaturated fatty acids, and rapid autolytic enzymatic degradation [303,304,305]. Spoilage in aquatic products proceeds through a well-characterized sequence: endogenous enzyme activity → nucleotide breakdown → biogenic amine accumulation → volatile sulfur compound release → overt putrefaction. IRN-based preservation must therefore address multiple simultaneous deterioration pathways.

Temperature-responsive IRNs are particularly valuable for aquatic products stored under strict cold chain conditions [306]. Phase-change materials (PCMs) encapsulated within SiO_2_ or polymer shells can absorb latent heat during brief temperature excursions, buffering against thermal abuse that would otherwise accelerate enzymatic and microbial spoilage. Concurrently, H_2_S-responsive colorimetric probes (e.g., MnO_2_/Au/Ag nanoframes [262]) excellent selectivity against thiol-type reductants and dimethyl sulfide (Figure 4C), providing sensitive evaluation of early-stage spoilage. The spoilage-responsive gas sensor is concentrated precisely where it is most needed (Figure 4D), minimizing unnecessary exposure and preserving the delicate flavor profile of fresh aquatic products.

### 5.4. Poultry Products

Poultry products are frequently threatened by high-risk foodborne pathogens such as *Salmonella* and *Listeria monocytogenes* on skin and muscle surfaces [307,308,309,310], posing severe public health concerns. To mitigate these risks, IRNs leverage active antimicrobial nano-coatings and essential oil nanoemulsions. These advanced systems provide targeted, stimulus-activated inhibition against surface contamination in response to microbial metabolites or localized pH shifts. Crucially, this localized and on-demand release mechanism ensures effective pathogen inactivation while preserving the sensory attributes, texture, and nutritional value of raw poultry products during cold-chain storage and distribution.

### 5.5. Dairy Products

Dairy products present unique preservation challenges driven by the competitive growth of starter cultures versus spoilage/pathogenic microorganisms [311,312,313], pH evolution, and lipid oxidation in high-fat products. In cheese [314,315], pH-responsive IRNs can visually track acidification and post-ripening alkalinization caused by surface mold growth. For fluid milk [316], antimicrobial nanocomposites releasing nisin or lysozyme in response to temperature fluctuations can suppress *Bacillus cereus* and *Pseudomonas* spp. without interfering with beneficial lactic acid bacteria. However, the high protein and calcium content of dairy matrices can induce nanoparticle aggregation or nonspecific protein corona formation, compromising responsive sensitivity, a challenge that remains underexplored in current IRN designs.

### 5.6. Bakery Products

Bakery products are highly susceptible to moisture migration [317,318], staling, and mold contamination. Humidity-responsive IRNs embedded in packaging liners can absorb excess moisture released during storage while releasing encapsulated propionic acid or essential oils when a_w_ exceeds critical thresholds. CO_2_-responsive indicators can detect respiratory activity of spoilage molds within sealed bread bags. Nevertheless, the low water activity of dry bakery items limits the mobility of responsive nanomaterials, often necessitating hygroscopic nanocarrier substrates that locally concentrate moisture for trigger activation.

### 5.7. Beverages

Beverages [319,320,321], including fruit juices, wine, and functional drinks, face microbial spoilage by acid-tolerant yeasts, molds, and acetic acid bacteria, alongside oxidative browning and flavor degradation. Light-responsive photocatalytic IRNs can generate ROS upon UV/visible exposure to sterilize liquid contents without thermal pasteurization. Gas-responsive caps or labels detecting CO_2_ accumulation from microbial fermentation provide early spoilage warnings. Critical limitations include the potential for photocatalytic off-flavor generation and the sedimentation of dense metal-oxide nanoparticles in low-viscosity beverages, which demands colloidally stable, neutrally buoyant nanoformulations that current research has not fully optimized.

Table 5 summarizes the tailored application of IRNs across different food categories. The comparative overview highlights how the selection of responsive mechanisms should align with specific deterioration pathways, aiming to achieve optimal preservation outcomes. The table thus serves as a practical reference for designing food-specific IRN systems that integrate sensing, antibacterial, and environmental regulation functions in a coordinated manner.

## 6. Active Prevention and Control

**Figure 5:**
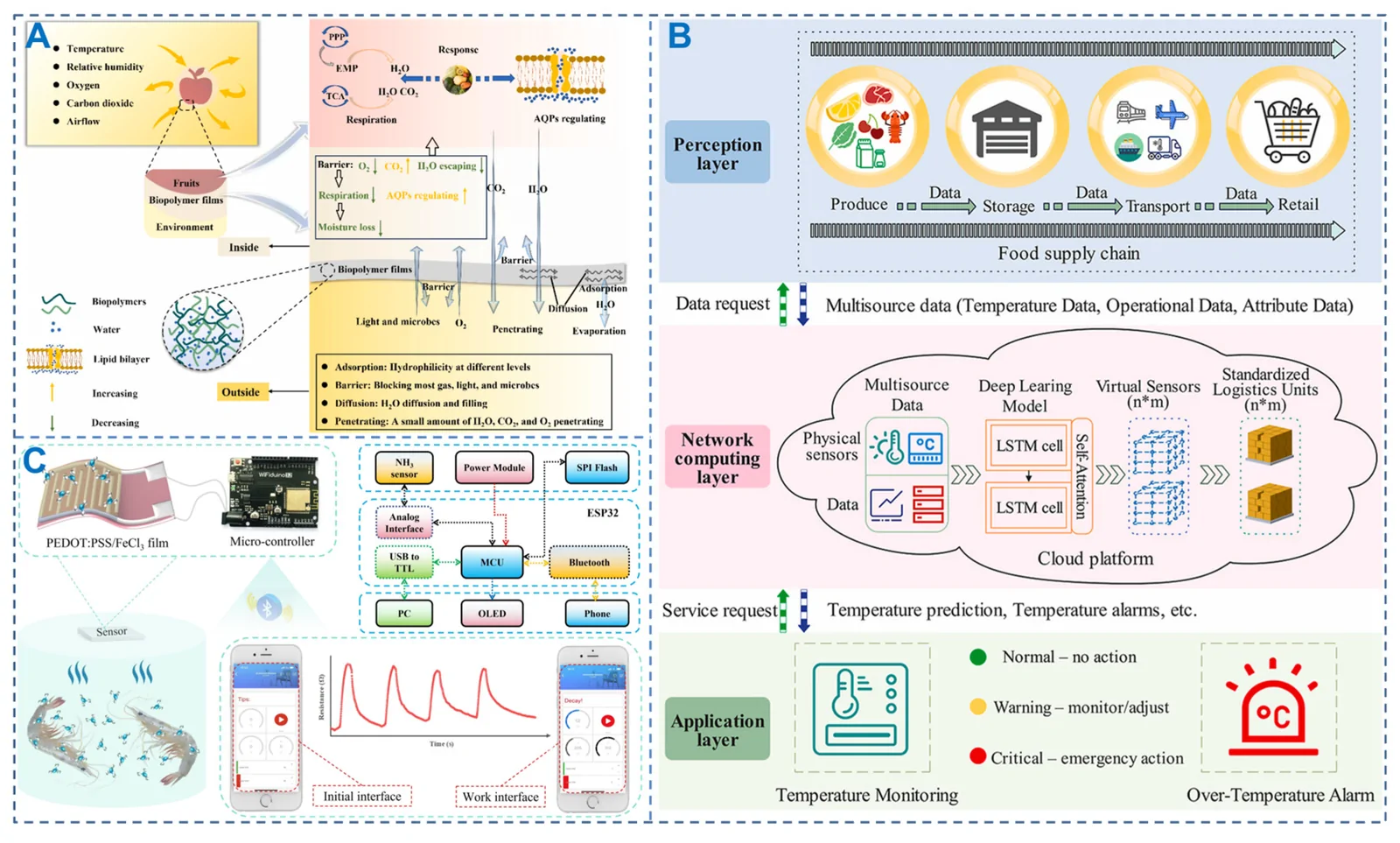
(**A**) Humidity control in fruits and vegetables packaging using bilayer asymmetric films. Reprinted with permission from Ref. [350]. Copyright 2025 Elsevier. (**B**) Data-driven virtual sensor systems for dynamic temperature monitoring along food supply chains. Reprinted from Ref. [351]. (**C**) Highly sensitive and flexible ammonia sensor based on PEDOT:PSS doped with Lewis acid for wireless food monitoring. Reprinted with permission from Ref. [352]. Copyright 2024 Elsevier.

Building upon the fundamental capabilities of freshness indication (Section 2), antibacterial mechanisms (Section 3), and environmental regulation (Section 4), the systems embed IRNs into practical food supply chain to create a practical framework that continuously monitors, predicts, and intervenes against spoilage risks (Figure 5).

### 6.1. Built-In Packaging Prevention and Control

A built-in packaging prevention and control integrates IRNs directly into food packaging materials [353,354,355,356], establishing a “packaging-food-environment” tripartite active prevention framework [357,358]. This system comprises three functionally interconnected components: a nano-sensing unit, an intelligent response unit, and a data transmission unit (Figure 5A).

The nano-sensing unit, typically composed of colorimetric or fluorescent IRN-based indicators, continuously detects spoilage biomarkers such as TVB-N [359], pH, C_2_H_4_, or biogenic amines. Upon detecting a predefined threshold indicative of incipient spoilage, the sensing signal activates the intelligent response unit, in which a reservoir of antibacterial agents are encapsulated within stimulus-responsive nanocarriers. For example, a pH-triggered release system containing AgNPs or natural antimicrobials (e.g., thymol-loaded mesoporous SiO_2_) can be embedded in the inner lining of the package; when pH rises due to microbial metabolism, the nanocarriers disintegrate and release their payload directly onto the food surface, suppressing pathogen proliferation at its onset. Simultaneously, the data transmission unit (comprising NFC tags or QR codes printed with conductive nano-inks) records the freshness status and transmits it to a cloud platform or consumer smartphone [360,361], enabling remote monitoring and supply chain transparency.

### 6.2. Cold Chain Integrated Prevention and Control

A cold chain integrated prevention and control deeply embeds IRNs within cold chain logistics equipment to construct an active prevention framework of “temperature regulation-quality monitoring-dynamic preservation” [362,363,364], ensuring food quality stability during transportation and storage. While conventional cold chain systems focus solely on maintaining set-point temperatures [365,366], they cannot compensate for inevitable temperature excursions caused by door openings, equipment failures, or uneven air circulation. IRN-enhanced cold chain systems address this gap by adding real-time quality sensing and adaptive intervention capabilities.

Temperature-responsive IRNs play a central role in this subsystem [367,368]. More advanced designs incorporate thermochromic IRNs that change color irreversibly upon exceeding a critical temperature threshold, providing a permanent visual record of cold chain breaches. Beyond passive buffering, active intervention can be achieved through integration with smart vents or gas-release actuators. Furthermore, temperature-logging IRN-based RFID tags enable continuous data collection (Figure 5B), feeding into predictive algorithms that estimate the remaining shelf life [369]. This integration transforms cold chain logistics from a static temperature-controlled corridor into an intelligent, self-regulating preservation ecosystem.

### 6.3. Traceability and Tracking Prevention and Control

A traceability and tracking prevention system integrates IRNs with Internet of Things (IoT) and blockchain technology to establish an active prevention framework of “source control-full chain monitoring-risk traceability” (Figure 5C). At the core of this system are IRN-based smart tags that combine freshness indication with wireless communication capabilities. For example, a label integrating anthocyanin-based colorimetric sensors with a printed NFC antenna can transmit both the current colorimetric reading and a time-stamped history of temperature and humidity exposures to a blockchain ledger. Each stakeholder along the supply chain can query the tag and verify the product’s quality trajectory. If a deviation from acceptable parameters is detected (e.g., a temperature excursion during transit that exceeds safe limits), the system automatically flags the affected batch and triggers a recall alert before the product reaches consumers. Moreover, machine learning algorithms trained on historical spoilage data can predict the remaining shelf life of individual packages [361], enabling dynamic pricing or prioritized distribution decisions.

## 7. Challenges and Future Directions

Despite the remarkable progress in developing IRN-based active preservation systems, several critical challenges must be addressed before these technologies can achieve widespread commercial adoption. The transition from laboratory prototypes to industrial-scale applications requires overcoming hurdles related to biosafety, multifunctional integration, intelligent system deployment, and environmental sustainability. A critical appraisal of current literature reveals that while IRNs exhibit remarkable laboratory-scale versatility, significant gaps remain between theoretical design and commercial translation. Past studies have predominantly focused on descriptive demonstrations of “sense-and-act” phenomena, frequently overlooking the intrinsic trade-offs inherent in these systems. For instance, the aggressive generation of ROS or rapid metal-ion release, while effective for pathogen inactivation, poses severe risks of collateral oxidative damage to food proteins and lipids, potentially compromising sensory and nutritional quality. Furthermore, the multi-step, complex fabrication protocols required for sophisticated hierarchical nanoplatforms suffer from prohibitive costs and poor batch-to-batch reproducibility, rendering them economically unviable for large-scale food packaging. Moving forward, the field must transition from empirical descriptions of novel nano-formulations to rigorous, mechanism-driven evaluations of real-world stability, chronic biosafety, and lifecycle environmental impacts.

### 7.1. Biosafety: Cytotoxicity, Migration, and Metabolism

Biosafety and regulatory compliance represent the most formidable obstacles to the industrial translation of IRNs in food preservation [370,371,372,373]. While acute cytotoxicity assays are standard, they are fundamentally inadequate for evaluating real-world scenarios characterized by chronic, low-dose ingestion through daily dietary intake. Prolonged exposure to engineered nanomaterials [374] poses significant risks of disrupting human intestinal epithelial barriers and inducing gut microbiota dysbiosis. Furthermore, current migration testing methodologies, designed primarily for conventional bulk plastics using static food simulants, fail to accurately capture the dynamic, stimulus-triggered release and degradation kinetics characteristic of IRNs.

From a regulatory standpoint, governing bodies such as the European Food Safety Authority (EFSA) and the U.S. Food and Drug Administration (FDA) enforce stringent pre-market safety assessments for nanomaterials in food contact applications, requiring comprehensive data on nanoparticle size distribution, agglomeration behavior, and systemic toxicokinetics. However, standardized protocols specifically tailored for stimuli-responsive nano-systems remain lacking. Beyond human health, the environmental lifecycle impact of accumulated residual nanoparticles in agricultural soils and aquatic ecosystems demands rigorous ecotoxicological scrutiny. Overcoming these barriers requires adopting a “safe-by-design” paradigm, coupling comprehensive in vivo toxicological profiling with transparent risk communication to foster consumer acceptance of nanotechnology-enabled food systems.

### 7.2. Multifunctional Integration and Precision

Integrating sensing, antibacterial, antioxidant, and gas-regulating functions into a single IRN platform is essential for comprehensive active preservation [375]. However, current designs face trade-offs between functional density and colloidal stability, as well as unintended cross-interference among modules. Future development should pursue “precision, intelligence, and greenness.” Precision involves programming orthogonal responsiveness to multiple spoilage biomarkers [292,376,377], enabling hierarchical decision-making (e.g., antioxidant release first, then antibacterial activation). Intelligence incorporates feedback loops that allow IRNs to adapt sensitivity [378] based on cumulative exposure history [379]. Greenness mandates the use of biocompatible or edible materials [380]. Advances in modular nanofabrication, computational modeling [381], and high-throughput screening will be critical to realize these goals.

### 7.3. Ai-Iot Enabled Intelligent Preservation

The convergence of artificial intelligence (AI) and IoT with IRN technology promises to transform food preservation into an autonomous, data-driven discipline [382,383,384]. Current systems rely on simple threshold alerts, but future platforms will employ machine learning models (e.g., recurrent neural networks, transformers) trained on multimodal sensor data to predict spoilage trajectories with high accuracy [385]. Edge computing will enable real-time inference on low-power microcontrollers attached to packaging, reducing latency and cloud dependence. Digital twin technology can simulate entire supply chains [53,386], allowing proactive optimization of preservation strategies.

While the integration of AI, IoT, and blockchain with IRNs offers a visionary paradigm for autonomous food preservation, many of these digital concepts currently remain largely forward-looking and lack widespread industrial implementation. Transitioning these smart platforms from laboratory proofs-of-concept to commercial reality requires overcoming severe techno-economic barriers. From a manufacturing standpoint, scaling up multi-step nano-functionalized films and printing conductive or responsive sensor arrays introduces prohibitive production costs and batch-to-batch reproducibility issues. Furthermore, ensuring seamless compatibility with existing high-speed industrial packaging technologies (such as thermoforming, modified atmosphere sealing, and automated labeling lines) presents major engineering challenges, as delicate nanostructures risk degradation under thermal and mechanical stress.

### 7.4. Green and Sustainable Nanomaterials

Developing green and sustainable nanomaterials is imperative to address environmental and biosafety concerns associated with conventional IRNs [387]. The guiding philosophy [388] is “derived from nature, returning to nature [389].” Key strategies include using renewable biomass feedstocks [390] as biodegradable nanocarriers [391]; employing green synthesis routes [392] that avoid toxic solvents; engineering stimuli-responsive degradation profiles so IRNs disassemble into harmless monomers after use; and adopting circular economy principles for composting or recycling spent packaging. Protein-based nanoparticles [393,394] and polyphenol–metal [395,396,397] coordination networks exemplify this direction [398], offering intrinsic antioxidant/antimicrobial activity alongside full biodegradability.

## 8. Conclusions

This review has systematically elucidated the transformative role of IRNs in advancing food preservation from passive containment to active, autonomous quality assurance. By synergizing freshness indication, hierarchical antibacterial mechanisms with intelligent environmental regulation, IRNs establish a closed-loop preservation framework that dynamically responds to spoilage signals. Tailored applications across different food systems demonstrate that matching responsive materials to specific deterioration pathways maximizes preservation efficacy. The integration of IRNs into packaging, cold chain logistics, and IoT-blockchain traceability platforms further enables real-time monitoring, predictive intervention, and full-chain accountability. Despite challenges in biosafety, multifunctional precision, and sustainability, the convergence of AI-driven analytics and green nanomaterial design promises to realize a new paradigm of “full-chain coverage, multi-dimensional synergy, and intelligent decision-making”.

## Figures and Tables

**Table 1:** Comparison of Recent Review Articles on Intelligent Packaging/Nanomaterials and the Contributions of the Present Review.

Ref. & Year	Main Focus/Scope	Limitations/Missing Aspects	Unique Contributions of the Present Review
Development of multifunctional metal–organic framework (MOF)-based nanofiller materials in food packaging: A comprehensive review (2024) [52]	Focuses on MOF-based nanofillers in food packaging.	Restricted to MOF materials; lacks comprehensive coverage of sensing-antibacterial synergy.	Integrates broader IRN platforms and couples sensing with multi-tier active intervention.
Advances in controlled-release packaging for food applications (2025) [38]	Reviews controlled-release packaging for food applications.	Concentrates mainly on release kinetics; lacks real-time visualization and microenvironmental regulation.	Highlights “sense-response-execution” synergy and smart environmental feedback.
Toward sustainable food packaging: innovations in biodegradable materials, smart technologies, and AI integration (2026) [53]	Discusses sustainable packaging, smart technologies, and AI integration.	Broad macroscopic perspective; lacks microscopic mechanistic insights into nano-antimicrobial pathways.	Bridges microscopic hierarchical antibacterial mechanisms with macroscopic AI-IoT cold chain logistics.
Application of Smart Packaging in Fruit and Vegetable Preservation: A Review (2025) [54]	Reviews smart packaging applications specifically for fruit and vegetable preservation.	Limited to produce; lacks cross-system comparisons (e.g., meat and aquatic products).	Provides a systematic, multi-food system comparative framework (fruits, meat, and aquatic products).
Present Review (2026)	Comprehensive synthesis of IRNs spanning freshness indication, hierarchical antibacterial action, smart environmental regulation, and AI-IoT supply chain integration.	— (This Work)	Establishes a holistic “sense-response-execution” closed-loop preservation paradigm with food-specific tailoring and AI-IoT future directions.

**Table 2:** Comparative Performance Matrix of Colorimetric versus Fluorescent Sensing Systems for Intelligent Food Packaging.

Sensing System Type	Detection Limits (LOD)	Response Time	Reversibility	Selectivity	Long-Term Stability	Commercial Feasibility
Colorimetric Systems (e.g., Halochromic dyes, Plasmonic metal nanoparticles) [98,99,100,101]	Moderate to High (typically ppm to percentage levels for volatile gases)	Rapid (Seconds to a few minutes; instant visual change)	Generally Irreversible (designed for single-use cumulative spoilage indication)	Moderate to High (can be tuned via specific chelators or reactive ligands)	Limited (prone to dye leaching, fading, and photo-oxidation over months)	High (Low cost, disposable, compatible with standard printing and naked-eye detection)
Fluorescent Systems (e.g., Carbon dots, Quantum dots, Organic fluorophores) [102,103]	Exceptionally Low (ppb to ppt levels; high sensitivity)	Fast to Moderate (Seconds to minutes, depending on diffusion and quenching kinetics)	Variable (some supramolecular or covalent systems offer partial or full reversibility)	High (ratiometric or dual-emission designs minimize background interference)	Moderate to Low (susceptible to photobleaching, quenching, and matrix degradation)	Moderate to Low (Requires portable readers/UV lamps, higher material costs, complex integration)

**Table 3:** Comparison of Physical, Chemical, and Biological Antibacterial Mechanisms of IRNs.

Feature	Physical Mechanism [126] 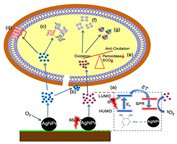	Chemical Mechanism [178] 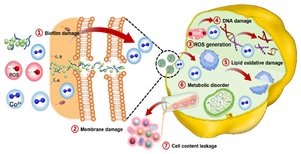	Biological Mechanism [179] 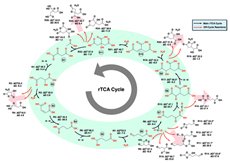
**Primary Target**	Cell wall/membrane integrity	Intracellular biomolecules (DNA, enzymes, proteins)	Signaling pathways (quorum sensing), metabolic enzymes
**Mode of Action**	Direct contact → membrane disruption, mechanical puncture	Ion release (Ag^+^, Zn^2+^, Cu^2+^) or ROS generation (•OH, O_2_^−^, H_2_O_2_)	Competitive inhibition of enzymes, QS signal degradation/interference
**Speed of Action**	Immediate (seconds to minutes)	Moderate (minutes to hours, depending on release kinetics)	Delayed (hours to days, affecting population-level behavior)
**Stimulus Responsiveness**	Low (mainly physical contact)	High (pH, temperature, light, enzyme-triggered release)	Medium (responsive to cell density or specific metabolites)
**Resistance Risk**	Very low (physical damage is difficult to circumvent)	Moderate (efflux pumps, detoxification enzymes may evolve)	Low (targets communication, not essential survival; reduced selective pressure)
**Advantages**	Rapid, broad-spectrum, no toxic chemicals needed	Sustained activity, controllable release, high potency	Highly specific, minimal side effects on food matrix, anti-biofilm
**Limitations**	Requires close contact; may be hindered by biofilm matrix	Potential metal ion migration, cytotoxicity at high doses	Slower effect; may not kill bacteria directly, only attenuate virulence
**Synergy with Other Mechanisms**	Creates membrane pores → facilitates ion/ROS entry; weakens biofilm → enhances QS inhibitor access	Oxidative stress damages DNA/enzymes → amplifies biological interference; ions disrupt QS signaling	Reduces biofilm formation → exposes deeper cells to physical/chemical attack; lowers resistance threshold

**Table 4:** Quantitative Performance Metrics of Representative IRNs in Food Packaging Applications.

IRN System/Core Material	Target Food Matrix/Spoilage Agent	Detection Limit (LOD)/Response Time	Antibacterial Efficiency (Log Reduction)	Release Kinetics/Trigger Mode	Shelf-Life Extension Rate	Nanoparticle Migration Level
Gas/Enzyme Sensors (e.g., MOF/Dyes) [236,237,238]	Meat/Volatile Amines (NH_3_, H_2_S)	LOD: 0.5 ppm; Response: 30–60 s	N/A (Sensing only)	N/A (Colorimetric optical shift)	N/A (Real-time monitoring)	Below regulatory limits (<2.0 mg/dm^2^ total migration)
Halochromic Nanocomposite Films (e.g., Cellulose/Anthocyanin) [239,240,241,242]	Seafood, Pork/Total Volatile Basic Nitrogen (TVB-N)	LOD: 1.2 ppm; Response: 10~30 min	N/A (Sensing only)	Diffusion-controlled dye retention	N/A (Freshness indication)	Minimal leaching (<3.5 mg/kg specific migration)
Nano-antimicrobials (e.g., Essential Oil/Dandelion Polysaccharide Nanofiber) [243,244]	Poultry/*Salmonella* & *Listeria*	N/A (Active preservation)	3.5~4.8 log reduction within 2 h	<5% baseline release at pH 6.5; >80% burst release at pH 5.0	80–150% extension over control	Ag/Zn ions well below EU limits (<0.05 mg/kg food)
Enzyme-Responsive Systems (e.g., chitosan/PEO) [245,246,247,248,249,250,251]	Fresh Pork/Lactic Acid & Spoilage Enzymes	N/A (Active preservation)	4.0~5.2 log reduction against *E. coli*	Zero-order sustained release triggered by microbial lipase/esterase	100%~200% extension (up to 14 days storage)	Negligible nanoparticle skeletal migration (<1 mg/kg)
Humidity/Temperature Buffering IRNs (e.g., Carboxymethyl cellulose/sodium alginate) [252,253,254,255,256,257,258,259]	Fruits & Vegetables/Respiration & Condensation	Response: 5~15 min (moisture/temp shift)	2.0~3.0 log reduction (mold spore inhibition)	Moisture-swelling controlled permeability	50%~120% reduction in weight loss and decay	Fully compliant with food-contact polymer migration tests

**Table 5:** IRN Applications Across Diverse Food Categories.

Food Category	Primary Preservation Challenges	IRN Trigger Mechanisms	Responsive & Active Mechanisms	Preservation Performance
**Fruits & Vegetables** 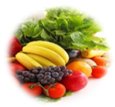	Post-harvest respiration [322,323], C_2_H_4_-driven ripening, moisture loss [324], fungal decay [325]	Gas-responsive (C_2_H_4_), Humidity-responsive	Porous MOFs for selective C_2_H_4_ adsorption; moisture-buffering nanocomposite films	Suppresses ripening [326], reduces weight loss, inhibits mold growth, extends post-harvest shelf-life
**Meat Products** 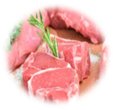	Lipid oxidation, protein degradation [327], microbial proliferation [328,329,330], off-odor accumulation [331]	pH-responsive, Enzyme-responsive	pH-triggered release of natural antimicrobials in response to initial lactic acid [140]; antioxidant nano-delivery [332,333]	Delays rancidity, suppresses total viable counts (TVC), maintains acceptable color and nutritional value
**Aquatic Products** 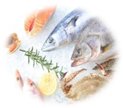	Autolysis, H_2_S/biogenic amine accumulation [334], nucleotide breakdown	Gas-responsive (H_2_S, volatile amines) [335], Temperature-responsive	PCM for cold-chain buffering [336]; highly sensitive H_2_S-responsive nanoframes [337]	Drastically lowers TVB-N, extends raw freshness, and provides real-time spoilage warning
**Poultry Products** 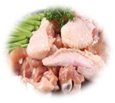	High-risk surface contamination (*Salmonella*, *Listeria*), rapid moisture-induced spoilage [338,339,340,341]	Enzyme-responsive, pH-responsive	Triggered release of essential oils or nano-silver/nano-zinc systems upon bacterial metabolite accumulation	Achieves targeted pathogen inactivation on surfaces without altering sensory profiles or causing cytotoxicity
**Dairy Products** 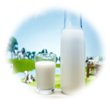	Psychrotrophic bacterial post-processing contamination [342], lipid oxidation, unwanted acidification	pH-responsive, Temperature-responsive	Active bionanocomposite films with localized antimicrobial release against microbial pockets	Prevents localized spoilage, controls curd/milk pH shifts, and maintains sensory flavor stability
**Bakery Products** 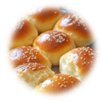	Mold/fungal growth, starch retrogradation (staling), and excess moisture migration [343,344,345]	Humidity-responsive, Enzyme-responsive	Moisture-regulating nanostructures combined with slow-release antifungal agents	Inhibits crumb mold formation, preserves softness and texture, and delays staling during extended storage
**Beverages** 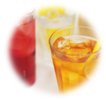	Thermal degradation, non-enzymatic browning, oxidation of sensitive bioactive components [346,347,348]	Light-responsive [349], Temperature-responsive	Nano-encapsulated antioxidants and responsive colloidal stabilization systems	Protects functional ingredients against light/heat stress, prevents oxidation, and ensures shelf-life clarity

## Data Availability

No new data were created or analyzed in this study. Data sharing is not applicable to this article.
